# Associations between antipsychotics-induced weight gain and brain networks of impulsivity

**DOI:** 10.1038/s41398-024-02881-4

**Published:** 2024-03-26

**Authors:** Claire Grosu, Paul Klauser, Daniella Dwir, Ines Khadimallah, Yasser Alemán-Gómez, Nermine Laaboub, Marianna Piras, Margot Fournier, Martin Preisig, Philippe Conus, Bogdan Draganski, Chin B. Eap

**Affiliations:** 1 Unit of Pharmacogenetics and Clinical Psychopharmacology, Centre for Psychiatric Neuroscience, Department of Psychiatry, Lausanne University Hospital and University of Lausanne, Prilly, Switzerland; 2 Service of Child and Adolescent Psychiatry, Department of Psychiatry, Lausanne University Hospital and University of Lausanne, Lausanne, Switzerland; 3 Center for Psychiatric Neuroscience, Department of Psychiatry, Lausanne University Hospital and University of Lausanne, Prilly, Switzerland; 4 Connectomics Lab, Department of Radiology, Lausanne University Hospital, Lausanne, Switzerland; 5 Psychiatric Epidemiology and Psychopathology Research Center, Department of Psychiatry, Lausanne University Hospital and University of Lausanne, Prilly, Switzerland; 6 Service of General Psychiatry, Department of Psychiatry, Lausanne University Hospital, Prilly, Switzerland; 7 Laboratory for Research in Neuroimaging LREN, Centre for Research in Neuroscience - Department of Clinical Neurosciences, Lausanne University Hospital and University of Lausanne, Lausanne, Switzerland; 8 Neurology Department, Max-Planck-Institute for Human Cognitive and Brain Sciences, Leipzig, Germany; 9 School of Pharmaceutical Sciences, University of Geneva, Geneva, Switzerland; 10 Center for Research and Innovation in Clinical Pharmaceutical Sciences, Lausanne University Hospital and University of Lausanne, Lausanne, Switzerland; 11 Institute of Pharmaceutical Sciences of Western Switzerland, University of Geneva and University of Lausanne, Lausanne, Switzerland

**Keywords:** Neuroscience, Schizophrenia

## Abstract

Given the unpredictable rapid onset and ubiquitous consequences of weight gain induced by antipsychotics, there is a pressing need to get insights into the underlying processes at the brain system level that will allow stratification of “at risk” patients. The pathophysiological hypothesis at hand is focused on brain networks governing impulsivity that are modulated by neuro-inflammatory processes. To this aim, we investigated brain anatomy and functional connectivity in patients with early psychosis (median age: 23 years, IQR = 21–27) using anthropometric data and magnetic resonance imaging acquired one month to one year after initiation of AP medication. Our analyses included 19 patients with high and rapid weight gain (i.e., ≥5% from baseline weight after one month) and 23 patients with low weight gain (i.e., <5% from baseline weight after one month). We replicated our analyses in young (26 years, IQR = 22–33, *N* = 102) and middle-aged (56 years, IQR = 51–62, *N* = 875) healthy individuals from the general population. In early psychosis patients, higher weight gain was associated with poor impulse control score (β = 1.35; *P* = 0.03). Here, the observed brain differences comprised nodes of impulsivity networks - reduced frontal lobe grey matter volume (*P*_corrected_ = 0.007) and higher striatal volume (*P*_corrected_ = 0.048) paralleled by disruption of fronto-striatal functional connectivity (*R* = −0.32; *P* = 0.04). Weight gain was associated with the inflammatory biomarker plasminogen activator inhibitor-1 (β = 4.9, *P* = 0.002). There was no significant association between increased BMI or weight gain and brain anatomy characteristics in both cohorts of young and middle-aged healthy individuals. Our findings support the notion of weight gain in treated psychotic patients associated with poor impulse control, impulsivity-related brain networks and chronic inflammation.

## Introduction

Current advances in studying the drivers of antipsychotics-induced weight gain and associated metabolic dysfunction in patients with mental disorders brought evidence of a plethora of potential mechanisms. There is strong empirical evidence about the role of underlying psychopathology, genetic factors, pharmacological treatment, and/or lifestyle factors (i.e., unhealthy diet and/or lack of physical activity) [1, 2]. Studies report a 2 to 3 fold increase in mortality rates among psychiatric patients compared with the general population, corresponding to a 10- to-15-year reduction in life expectancy [3]. Two-thirds of the increased mortality risk is attributed to cardiovascular disease [4]. Patients treated with psychotropic drugs (all antipsychotics, some antidepressants and mood stabilizers) frequently show disproportional weight gain, which can affect their psychological well-being leading to treatment interruption and to a relapse of the illness [5]. Additionally, there is the notion that antipsychotics change the appetite regulation (i.e., excessive food consumption) with a large interindividual variation in the susceptibility to such effects [6, 7].

Obesity [8–11], binge eating disorder [12] and food addiction [8, 13] are associated with impaired impulse control. Grey matter (GM) volume loss and neural processing biases across network nodes involved in impulse inhibition, such as the striatum and frontal lobe, are among the most frequent observations in structural and functional imaging studies in overweight and obese participants [14–21]. Along these lines, also drug-naïve patients experiencing their first psychotic episode [22–25] and pharmacologically treated patients with schizophrenia [26–29], show differences in GM volume and structural brain connectivity.

Up to date, there are only a handful of studies that have examined the associations between brain morphology or function and metabolic changes induced by antipsychotics [30, 31]. One region-of-interest study reported a higher striatal volume and decreased striatal functional connectivity that correlated with weight gain in 81 early-phase psychosis patients treated for 12 weeks with risperidone or aripiprazole [30]. With reference to brain activation related to presentation of food stimuli, another study showed that olanzapine treatment enhanced both the anticipatory and consummatory reward responses to food [31]. A decrease in responsivity to food consumption in areas associated with inhibition of feeding behaviour was also noted [31].

Recent evidence in patients with schizophrenia supports the notion of vulnerability due to interaction between aberrant inflammatory response and the presence of metabolic syndrome. Plasminogen activator inhibitor-1 (PAI-1) is the principal inhibitor of tissue plasminogen activator (tPA) and urokinase, and is therefore an inhibitor of fibrinolysis [32]. High plasma levels of PAI-1 have been associated with an increased risk of suffering from cardiovascular disease [33]. Moreover, pathways depending on PAI-1 are also thought to play a role in the development of obesity, insulin resistance and type 2 diabetes [34]. Interestingly, the *SERPINE-1* gene, which encodes for PAI-1 is overexpressed in the monocytes of patients with schizophrenia [35]. Macrophage migration inhibitory factor (MIF) is a pleiotropic cytokine involved in the regulation of innate and adaptive immunity [36] and higher levels of MIF were found in metabolic disease [37]. Normal MIF expression was found to be linked to metabolic dysfunction and insulin resistance induced by olanzapine, when compared to low MIF expression [38]. Several studies have also identified MIF level as a potential biomarker for schizophrenia [39–41]. These findings regarding PAI-1 and MIF levels lead to the hypothesis that they may be involved in the difference in weight gain in schizophrenia.

A plethora of factors could account for the interindividual variability in weight gain among patients treated with antipsychotics; however, the association with impulsivity, brain structure and function has not been clearly established yet. Here, we first sought to investigate, in a cohort of patients in the early phase of psychosis (Early Psychosis Patients; EPP), the associations between interindividual variability in weight gain following the introduction of antipsychotics and the structural and functional brain characteristics. We predicted that impulsivity and the anatomical and functional properties of brain regions which are relevant to impulsivity control would be linked to weight gain (i.e., frontal lobe and striatum). Additionally, we aimed to determine whether patients who had put on more weight had increased plasma levels of PAI-1 and/or MIF. By repeating these analyses in both a cohort of healthy individuals (cohort A) and a population-based cohort (cohort B), we sought to determine whether the observed associations were specific to EPP treated with weight gain-inducing antipsychotics.

## Methods

### Subjects

#### Early psychosis cohort

EPP (i.e., illness duration <5 years) were recruited from the Treatment and Early Intervention in Psychosis Programme (TIPP) [42] and from the PsyMetab cohort [43].

EPP within the first 3 years of treatment for a psychotic disorder and having met psychosis threshold according to the Comprehensive Assessment of At Risk Mental States criteria [44] were selected. A total of 42 patients who had an available brain magnetic resonance imaging (MRI) scan (during the first year after the introduction of antipsychotics) and who had an assessment of impulsivity were included in the current study. Based on previous findings from our group, patients were classified into a high (HW_EPP_, *N* = 23) or low (LW_EPP_, *N* = 19) weight gain group if they gained more than 5% or less than 5% of their initial weight after one month of antipsychotic treatment, respectively [45]. Patients included in the PsyMetab and TIPP cohorts gave their written informed consent to participate in the studies. PsyMetab and TIPP protocols were approved by the local Ethics Committee.

#### Cohort A

A total of 102 healthy psychotropic-naïve participants with no history of psychotic or substance use disorders were recruited from the same geographic area as in the TIPP programme. Only body mass index (BMI) was available for cohort A. Therefore, groups were formed based on participants’ BMI, with 83 classified as normal BMI < 25 kg/m2 and 19 as high BMI ≥ 25 kg/m2. Informed written consent in accordance with the institutional guidelines was obtained for all participants.

#### Cohort B

CoLaus|PsyCoLaus is a prospective cohort study designed to investigate cardiovascular risk factors and mental disorders as well as their associations in the community. A total of 6734 individuals aged 35 to 75 years were randomly selected according to the civil register of the city of Lausanne, Switzerland, between 2003 and 2006 and underwent a physical [46, 47] and psychiatric evaluation [48]. Since the baseline assessment, three follow-up evaluations were completed which took place from 2009 to 2013 (first follow up or F1), 2014 to 2018 (second follow up or F2) and 2018 to 2021 (third follow up or F3). The present analyses included data from the F1 and F2 evaluations. Computational brain anatomy analyses were confined to participants who accepted an MRI exam (BrainLaus, *N* = 1145). For the present study, the included participants (*N* = 875) were classified as follows, based on weight gained from the first to the second follow-up. Given the larger interval between two measures in cohort B, a 7% weight gain criterion was applied [49]: (i) the high weight gain group (HW_cohortB_, *N* = 729) if they had a weight gain of ≥7% [49] or more during the period between the first and second follow-up, and (ii) the low weight gain group (LW_cohortB_, *N* = 146) if they gained <7%. A total of 98 participants with weight loss >−7% and 172 with age ≥65 years of age at the first follow-up exam were excluded to avoid brain changes due to significant weight loss [50–52] and aging [53–57]. All participants gave written informed consent, and the study was approved by the local Institutional Ethics Committee.

### Clinical assessments

In EPP, weight measurements were completed at baseline, after one month of treatment, and at the time of MRI scanning. Cohort A weight was assessed only once during an interview. The consumption of cannabis was assessed with the Case Manager Rating Scale (CMRS) [58] in both EPP and cohort A. In cohort B, measures of weight, BMI and smoking were collected from standardized interviews and anthropometric assessments resulting in a comprehensive set indicative of disease history and cardiovascular risk [46].

Psychopathology and functional levels were scored with the Global Assessment of Functioning (GAF) scale, the Positive and Negative Syndrome Scale (PANSS) [59] and the Montgomery-Åsberg Depression Rating Scale (MADRS) [60].

Impulsivity in EPP was assessed in the early psychosis cohort using the PANSS score for “poor impulse control (G14)” [61]. This item assesses the degree of impulsivity on a scale from 1 to 7 (1 being the absence of impulsivity and 7 an extreme level of impulsivity). Duration of illness (DOI) was defined as the time between reaching the psychosis threshold for the first time and the time of assessment. Poor impulse control score was not available in cohort A. In cohort B, impulsivity control score was constructed using the Neuroticism and the Conscientiousness factors of the NEO-Five-Factor Inventory-Revised (NEO-FFI-R) [62] completed at F1. Impulsivity control scores were calculated as the square root of the sum of the squares of the normalized scores for Neuroticism and the normalized inversed scores for Conscientiousness.

Regarding plasma analyses, blood samples were subjected to two rounds of centrifugation. Firstly, a 10-min spin at 400 g and 4 °C was performed, and the resulting supernatant was collected in a fresh falcon tube. Secondly, the tube was centrifuged again at 3000 g for 10 min at 4 °C, and 500 μl aliquots were prepared. These aliquots were subsequently frozen at −80 °C until PAI-1 and MIF analysis. Plasma levels of PAI-1 and MIF were measured using ELISA kits (-ab157528, Abcam’s PAI-1 ELISA kits, Abcam, Cambridge, MA, USA; Human Active MIF ELISA Kit, R&D System, Abingdon, United Kingdom). All assays were performed according to the manufacturer’s instructions in EPP and cohort A.

### MRI acquisition and analysis

In both EPP and cohort A, MRIs were performed with 3 Tesla magnetic resonance scanner (Siemens Medical Solutions, Erlangen, Germany) equipped with 32-channel head coil. Each scanning session included a magnetization-prepared rapid acquisition gradient echo (MPRAGE) T1-weighted sequence and a 9-minute gradient echo-planar imaging (EPI) sequence that was sensitive to BOLD (blood-oxygen-level-dependent) contrast. The MPRAGE acquisition exhibited a 1 mm in-plane resolution and 1.2 mm slice thickness, encompassing 240 × 257 × 160 voxels (TR = 2.30 ms, TE = 2.98 ms, and TI = 900 ms). In contrast, the functional MRI (EPI) acquisition employed an isotropic 3.3 mm voxel size, with a 0.3 mm inter-slice gap, covering a total of 64 × 58 × 32 voxels (TR = 1920 ms and TE = 30 ms). During the resting-state fMRI (rs-fMRI) recordings, patients were instructed to lie calmly in the scanner with their eyes open, without fixating on any specific thought. The rs-fMRI sequence was initiated at the beginning of the session, immediately following the acquisition of the structural scan, with the accompaniment of an experienced psychologist for all patients throughout the scanning process. Ultimately, the acquisition procedure yielded a sequence of 280 BOLD images for each participant.

During the study, there was a routine MRI-system upgrade from the MAGNETOM-Trio to the MAGNETOM-Prisma system. Imaging parameters were precisely matched before and after the upgrade, and the same 32-channel head coil was used.

In order to obtain the sample size for a robust study and avoid scan bias, the MRI data was harmonized using the empirical Bayes approach ComBat - Combining Batches [63]. The global network analyses were repeated on data acquired on the MAGNETOM-Trio or on the MAGNETOM-Prisma system only to further exclude major effects of the scanner upgrade on the results. Additional information on the scanner effect could be found in supplementary material.

fMRI data were processed according to a state-of-the-art pipeline that involved several steps. These steps included the removal of the initial 5 time points to ensure signal stability, addressing slice-timing discrepancies, correcting for motion artifacts by regressing out six motion parameters, averaging signals from white matter and cerebrospinal fluid, performing linear detrending, and applying bandpass filtering within the frequency range of 0.01–0.1 Hz. All of these processing steps were carried out using the CMTK software [64]. Time series have been averaged over the Freesurfer (v.6.0.0, https://surfer.nmr.mgh.harvard.edu/) cortical regions. Functional connectivity has been obtained by computing the Pearson’s correlation between the mean temporal signals from each pair of brain regions. Four regions of interest have been selected: the inferior frontal gyrus, the putamen, the pallidum, and the primary motor cortex.

All imaging data for cohort B were acquired on the very same 3 T whole‐body MRI system (MAGNETOM Prisma; Siemens Medical Systems, Erlangen, Germany) using a 64‐channel radiofrequency receive head coil and body coil for transmission. Methods regarding the acquisition of cohort B were described somewhere else [65]. We sampled regional volume average values in individuals’ native space using factorization-based image labelling [66] after performing automated tissue classification using the multi-channel option of SPM12s “unified segmentation”. Aiming to adjust all regional values for the global effect of head size, we estimated its proxy – the total intracranial volume (TIV) from the sum of GM, WM, and CSF volumes [67].

### Statistical analysis

Descriptive data are shown as numbers and percentages for categorical variables or median and interquartile range (IQR) for continuous variables. To compare between groups, Wilcoxon-Mann-Whitney rank-sum test or the Chi-squared test were used, depending on the variable type.

The association between weight gain and the poor impulse control score was tested using linear regression model adjusted for covariates (age, sex, smoker status and weight at baseline for EPP or at F1 for cohort B).

Weight gain differences (HW_EPP_ vs. LW_EPP_ or HW_cohortB_ vs. LW_cohortB_) or the correlation between weight gain (%) for EPP, or BMI (<25 kg/m^2^ vs. ≥25 kg/m^2^) for cohort A, and brain volumes of region of interest (ROI), were independently tested using the general linear model at each voxel and the multiple regression analyses respectively, as implemented in Randomise (http://fsl.fmrib.ox.ac.uk/). Age, gender, and TIV were set as nuisance factors in the model. All results were corrected for multiple comparison Type I error with a non-parametric cluster-size based procedure [68, 69].

In cohort B, the associations between weight gain group or BMI status (<25 vs. ≥25 kg/m^2^) and GM volumes were tested with general linear regression analyses, adjusted for age, sex, and TIV.

The relationship between weight gain (%) and the fronto-striatal functional connectivity (RSFC) (i.e., the inferior frontal gyrus, putamen, pallidum and primary motor cortex) was assessed with the linear regression model in EPP and in cohort A. RSFC values were converted into Z-scores.

For inflammatory biomarker analyses, linear models were used to examine the association between plasma levels of PAI-1, or MIF and weight gain (%) in the EPP cohort, or BMI in the cohort A, adjusted for age and sex. Associations between inflammatory biomarkers and poor impulse scores were analysed. Considering that inflammatory markers have been associated with impulsive behaviour [70–72], we sought to investigate whether there is an association between PAI-1 or MIF levels and poor impulse control.

Analyses were performed in R (version 4.0.2; RStudio, Inc; Boston, Massachusetts). The statistical significance was set at a *P* ≤ 0.05.

## Results

### Study demographics

Among 42 EPP, high (≥5%) and low (<5%) weight gain were reported for 19 and 23 patients, respectively. Both groups (Table 1) were of similar age and sex (HW_EPP_: 37%; LW_EPP_: 35% women) and did not differ in terms of illness duration or in terms of treatment. There was no difference between the groups in the PANSS negative, PANSS positive and PANSS general scores, nor for total MADRS score or GAF score. EPP were younger than cohort A (*N* = 102) with a median age of 23 years (IQR: 21–27) versus 26 years (IQR: 22–33) for cohort A (*P* = 0.002). EPP had a lower level of education (13 years vs. 16 years; *P* < 0.001) and were more likely to smoke (59% vs. 8%; *P* < 0.001) and to use cannabis (29% vs. 5%; *P* < 0.001) when compared to cohort A (Supplementary Table 1).

**Table 1:** Demographic and clinical characteristics of early psychosis patients (*N* = 42).

	HW_EPP_ (*N* = 19)	LW_EPP_ (*N* = 23)	*P*^a^
**Women,** ***N*** **(%)**	7 (37%)	8 (35%)	1
**Age median (IQR), years**	24 (21–27)	22 (21–27)	0.33
**Weight at baseline median (IQR), kg**	69 (63–75)	71 (60–74)	0.63
**BMI at baseline median (IQR), kg/m²**	24 (20–25)	22 (21–24)	0.49
**Duration of illness median (IQR), years**^**b**^	0.77 (0.42–1.4)	0.77 (0.54–2.0)	0.63
**Years of education median (IQR), years**	13 (12–16)	12 (10–14)	0.33
**Years of education father median (IQR), years**	14 (9–16)	12 (9–17)	0.84
**Years of education mother median (IQR), years**	12 (9–15)	12(8–12)	0.44
**Cannabis users,** ***N*** **(%)**	6 (32%)	6 (27%)	0.96
**Smoking,** ***N*** **(%)**	11 (58%)	14 (61%)	1
**Medication:**			
Amisulpride	4 (21%)	6 (26%)	0.45
Aripiprazole	4 (21%)	2 (8.7%)	
Lurasidone	1 (5.3%)	0 (0%)	
Olanzapine	1 (5.3%)	2 (8.7%)	
Quetiapine	8 (42%)	8 (35%)	
Risperidone	1 (5%)	5 (22%)	
**PANSS positive score median (IQR)**	12 (11–16)	13 (9.5–15)	0.75
**PANSS negative score median (IQR)**	16 (13–18)	14 (11–23)	0.93
**PANSS general score median (IQR)**	33 (27–40)	32 (24–36)	0.68
**GAF score median (IQR)**	50 (45–68)	48 (41–59)	0.42
**MADRS total score median (IQR)**	12 (7.0–16)	13 (5.0–19)	0.94

Data are medians (IQR) or numbers (percentage).

*BMI* body mass index, *HW*_*EPP*_ high weight gain group, *IQR* interquartile range, *LW*_*EPP*_ low weight gain group, *MADRS* Montgomery-Åsberg depression rating scale, *PANSS* positive and negative syndrome scale.

^a^*P*-values for statistical comparisons between HW_**EPP**_/LW_**EPP**_, Wilcoxon–Mann–Whitney rank-sum tests for continuous variables and chi-square test for categorical variable; *P* < 0.05 in bold.

^b^Duration of illness at the time of the magnetic resonance imaging, defined as the temporal lapse (years) between the crossing of psychosis threshold (according to the Comprehensive Assessment of At Risk Mental States) and the date of the magnetic resonance imaging.

Within cohort A, there were no statistical differences for sex, smoking status and cannabis use between the high (≥25 kg/m²; *N* = 19) and normal BMI groups (<25 kg/m²; *N* = 83). There were differences in age, level of education and BMI between the two groups (Supplementary Table 2).

In cohort B, which included middle-aged individuals (*N* = 875, median 51 years, IQR: 46–57), there were differences for age (median 48 vs. 51 years, respectively; *P* < 0.001), sex (60% vs. 49% women; *P* = 0.01), and BMI during follow-up 2 (median 29 vs. 25 kg/m^2^; *P* < 0.001; Supplementary Table 3) between HW_cohortB_ and LW_cohortB_.

### Association between the poor impulse control score and weight gain

In the EPP, higher weight gain was significantly associated with a poor impulse control when corrected by age, sex, baseline weight and cigarette smoker status (β = 1.35, *P* = 0.03; Table 2). In cohort B, there was no association between the impulsivity control score at F1 and weight gain between F1 and F2 (β = 0.01, 95%CI = −0.36–0.38, *P* = 0.94, data not shown), when corrected by age, sex, smoker status and weight at F1.

**Table 2:** Associations between poor impulse control scores and weight gain (%) in early psychosis patients (*N* = 42).

	Weight gain (%)		
Predictors	*β*	95%CI	*P*
(Intercept)	0.61	−10.52–11.73	0.91
**Age (years)**	0.07	−0.17–0.30	0.57
**Women**	0.42	−2.36–3.20	0.76
**Smoking status (yes)**	−1.54	−3.83–0.74	0.18
**Weight at baseline (kg)**	0.01	−0.12–0.14	0.86
**Poor impulse control scores (PANSS G14)**	1.35	0.13–2.56	**0.03**

After correcting for age, sex and smoking status, weight gain was associated with poor impulse control scores. Estimates with 95% confidence intervals (CI) and *P*-values are reported from linear regression models. *P* < 0.05 in bold.

*β* beta coefficient, *CI* confidence interval, *PANSS G14* positive and negative syndrome scale, poor impulse control score.

### Differences in brain regions related to impulsivity

**Fig. 1:**
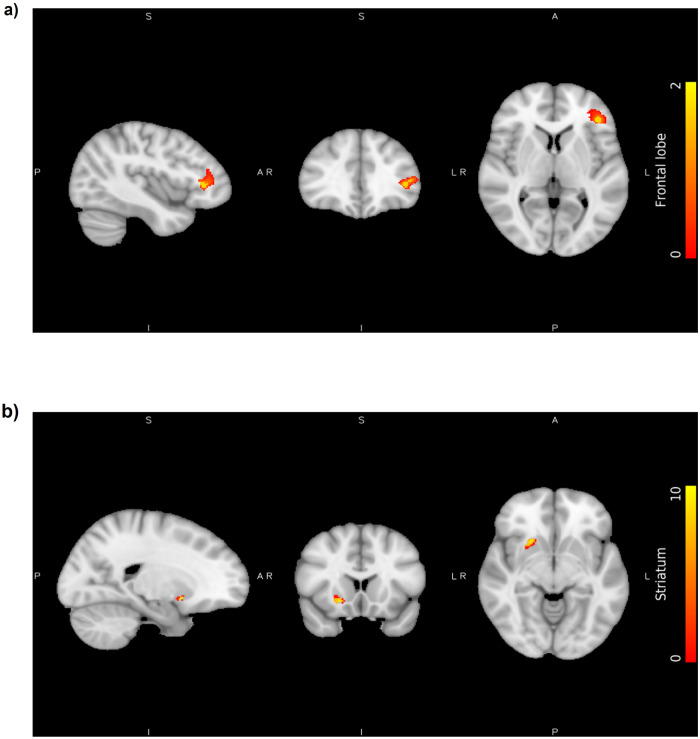
Volume differences in early psychosis patients (*N* = 42). **a** The clusters of voxels result from the comparison between the high weight gain group (HW_EPP_) and the low weight gain group (LW_EPP_) in the frontal lobe: the orange cluster located on the left frontal lobe represents areas of lower grey matter volume in HW_EPP_ (contrast: HW_EPP_ < LW_EPP_, MNI: 132,161,76*, P*_*corrected*_ = 0.007). The colour bar representing the *t* statistic. **b** The clusters of voxels result from the correlation between weight gain and volume of grey matter in the striatum: the yellow cluster located on the right striatum represents areas of higher grey matter volume (MNI: 69,142,65*, P*_*corrected*_ = 0.048). The colour bar represents the *t* statistic.

In the EPP, spatial clusters of significantly reduced GM in the frontal lobe (*P*_corrected_ = 0.007) were found in the HW_EPP_ group (*N* = 19) compared to the LW_EPP_ group (*N* = 23; Fig. 1a). No significant GM volume group difference was found in the striatum. Considering the whole early psychosis group, a positive correlation was identified by multiple regression analysis between the GM volume of the striatum and weight gain (%) after 1 month of treatment (*P*_corrected_ = 0.048; Fig. 1b).

As no longitudinal data are available for cohort A, associations were examined between brain structures and functions with BMI at the time of the scan (normal BMI < 25 kg/m² vs. high BMI ≥ 25 kg/m²). No associations based on BMI (continuous and dichotomous), or ROIs (data not shown) were found.

No association was found in cohort B, nor between HW_cohortB_ and the frontal lobe or the striatum volumes (Supplementary Table 4). When analysing participants according to BMI groups (BMI < 25 kg/m² vs. BMI ≥ 25 kg/m²), no statistical differences nor associations were shown with impulsivity related brain regions (data not shown).

### Correlation between fronto-striatal RSFC and weight gain

Pearson correlation was performed with the RSFC between the areas of the fronto-striatal circuit areas. In the early psychosis group, we observed a negative correlation between weight gain (%) and the RSFC of the right primary motor cortex to the pallidum (*R* = −0.32; *P* = 0.04; Supplementary Fig. 1). Considering the whole cohort A, no correlations were observed between BMI and RSFC (data not shown).

### PAI-1 and MIF markers

In EPP, higher PAI-1 levels were measured in the HW_EPP_ (*N* = 11) as compared to the LW_EPP_ (*N* = 15) group (34 vs. 14 U/mL; *P* = 0.02; Table 3). In linear models, after correction for age and sex, weight gain was associated with an increase in PAI-1 levels (*β* = 4.9; *P* = 0.002; Table 4). This association was absent for MIF levels (*N* = 12 for HW_EPP_, *N* = 12 for LW_EPP;_ *P* = 0.66). The association between PAI-1 and poor impulse control was investigated, however, no significant associations were found.

**Table 3:** Macrophage migration inhibitory factor and plasminogen activator inhibitor-1 levels in early psychosis patients and in the cohort A.

	EPP			Cohort A			Total EPP	Total cohort A	*P*^a^
	HW_EPP_	LW_EPP_	*P*^b^	Normal BMI	High BMI	*P*^c^			
MIF levels^d^	69 (45–110)	49 (34–62)	0.2	46 (36–69)	46 (34–53)	0.61	56 (34–89)	46 (35–66)	0.25
PAI-1 levels^d^	34 (21–67)	14 (4.9–27)	**0.02**	3.5 (1.9–9.6)	13 (5.2–28)	**0.04**	20 (6.0–35)	4.0 (2.1–11)	**<0.001**

*BMI* body mass index, *EPP* early psychosis patients, *HW*_*EPP*_ high weight gain group, *IQR* interquartile range, *LW*_*EPP*_ low weight gain group, *MIF* macrophage migration inhibitory factor, *PAI-1* plasminogen activator inhibitor-1.

*P* < 0.05 in bold.

^a^*P*-values for statistical comparison between Total EPP/Total cohort A. MIF: Data were not available for 18 out of 42 EPP patients and 32 out of 102 participants of cohort A. PAI-1: Data were not available for 16 out of 42 EP patients and 32 out of 102 participants of cohort A.

^b^*P*-values for statistical comparison between HW/LW from the EPP. MIF: Data were not available for 7 out of 19 HW patients and 11 out of 23 LW patients. PAI-1: Data were not available for 8 out of 19 HW patients and 8 out of 23 LW patients.

^c^*P*-values for statistical comparisons between Normal BMI group/High BMI group from cohort A. MIF: Data were not available for 24 out of 83 participants with normal BMI and 8 out of 19 participants with high BMI. PAI-1: Data were not available for 22 out of 83 participants with normal BMI and 8 out of 19 participants with high BMI.

^d^Data are medians (IQR), MIF levels are expressed in ng/mL and PAI-1 levels in U/mL.

**Table 4:** Linear models for Macrophage migration inhibitory factor and plasminogen activator inhibitor-1 levels in early psychosis patients (*N* = 42).

	MIF (ng/mL)			PAI-1 (U/mL)		
Predictors	*β*	95% CI	*P*	*β*	95% CI	*P*
**(Intercept)**	−31	−177–115	0.66	24	−27–75	0.34
**Age (years)**	3.9	−1.6–9.4	0.15	−0.90	−2.9–1.1	0.36
**Women**	−4.2	−52–44	0.86	9.3	−8.9–27	0.30
**Weight gain (%)**	1.7	−6.1–9.4	0.66	4.9	2.0–7.7	**0.002**

Estimates with 95% confidence intervals (CI) and *P*-values are reported from linear regression models. *P* < 0.05 in bold.

*β* beta coefficient, *BMI* body mass index, *MIF* macrophage migration inhibitory factor, *PAI-1* plasminogen activator inhibitor-1, *PAI-1* plasminogen activator inhibitor-1.

In cohort A, a difference in PAI-1 levels was observed between low and high BMI groups (3.5 vs. 13 U/mL; *P* = 0.04; Table 3). On the other hand, after adjustment for age and sex, BMI was not associated with PAI-1 or MIF levels in linear models (Supplementary Table 5).

Higher PAI-1 levels were measured in EPP as compared to subjects from cohort A (20 vs. 4.0 U/mL; *P* < 0.001; Table 3), and no difference in MIF levels was observed between EPP and cohort A.

The association between PAI-1 or MIF and poor impulse control was investigated, but no significant associations were found.

## Discussion

Our study on early psychosis patients treated with antipsychotics confirms associations between weight gain and poor impulse control, brain networks governing impulsivity and chronic inflammation.

In our study, we show that EPP treated with antipsychotics, the high and rapid weight gain was associated with poor impulsive control. This suggests that patients with increased impulsiveness have difficulties controlling their eating behaviour. Such an association was not found in the population-based cohort B, which suggests a specific mechanism in EPP. Then, by analysing brain regions related to impulsivity and reward, a decrease in frontal lobe GM volumes in the HW_EPP_ gain group was observed, as well as a positive correlation between weight gain and the striatum GM volume. These results are consistent with the previously reported decreased frontal GM volume following antipsychotic therapy [73] and reported positive correlation between striatal [30] volume and weight gain induced by antipsychotic treatment. The absence of frontal and striatal volume changes in cohort A and cohort B suggests that changes of volume in these regions are specific to weight gain in the early psychosis cohort. Altogether, the present results support the hypothesis that brain region related to impulsivity and reward, namely fronto-striatal structures, and poor impulse control scores are associated with weight gain induced by antipsychotics.

Our study extends previous findings by additionally investigating the properties of fronto-striatal connectivity. A negative correlation between weight gain and RSFC in the fronto-striatal circuits in the EPP group was identified. A functional MRI study in healthy volunteers exposed to one-week treatment with olanzapine showed increased responses to cues predicting rewarding liquids, while activations of striatum activities elicited by the image of tasteless liquid decreased. This suggests that striatum activity may be one of the mechanisms leading to weight gain induced by antipsychotics [31]. Of note, no correlation between BMI and RSFC in the fronto-striatal circuits was found in healthy individuals from cohort A. In patients with schizophrenia, there appears to be a significant association between emotion-related impulsivity (positive and negative emotions) and brain connectivity. In particular, one study has shown that schizophrenia patients with higher levels of emotion-related impulsivity have reduced connectivity between the ventral prefrontal and limbic/cognitive control regions and within the ventral prefrontal areas [74]. In contrast, increased connectivity has been observed between emotion-related impulsivity and sensory regions such as the middle occipital gyrus [74]. It is also worth noting that this issue extends beyond schizophrenia, as similar patterns have been observed in conditions such as binge eating disorder, where greater impulsivity in relation to negative emotions has been found [75]. Future research should examine these multiple dimensions of impulsivity in psychotropic drug-induced weight gain in patients with early psychosis.

The present study also sought to investigate whether PAI-1 and MIF levels, as markers of inflammation, were elevated in patients with higher weight gain. In the EPP, PAI-1 levels were significantly higher in patients who had significant weight gain after one month of treatment. Prospective studies have shown that PAI-1 is a predictor for future cardiovascular events [76, 77], and this is in line with a previous study that predicted cardiovascular events in patients who gained more than 5% of their baseline weight after 1 month of the introduction of an AP at risk [45]. The present study did not identify an association between MIF levels and changes in weight, contrary to a previous study that identified such an association [38]. The previous study took into account MIF expression, whereas our study just examined plasma analyses, which could be one reason for the discrepancy. In contrast to previous studies [39–41], no difference was also noted between MIF in EPP and cohort A. In one study, there was a difference in MIF levels between controls and patients who were naïve or not treated with AP for more than 6 weeks prior to sample collection [39]. Our analyses included patients who were being treated with AP drugs, which could explain the contradictory results. In addition, one study with older participants, including 86 patients with schizophrenia (mean age ± SD, 54.3 ± 10.3 years) and 51 controls (48.4 ± 9.5 years), observed that MIF levels were unrelated to the schizophrenia group when compared to the control group [41] after regression analyses, which is consistent with our findings. Although no significant association was found between PAI-1 or MIF levels and poor impulse control, it is important to note that the limited number of patients with available PAI-1 or MIF samples may have contributed to these null findings. In addition, the nature of the inflammatory markers and the multidimensional aspect of the impulsivity scores may have influenced our results compared to previous studies. Future research, with larger patient cohorts and specific facets of impulsivity [78], may provide a more comprehensive exploration of potential associations with PAI-1 or MIF.

Some limitations of our study must be acknowledged. First, results are limited by the cross-sectional design of the study, the MR scan being available only after the introduction of the antipsychotic. Further longitudinal studies are required to elucidate whether or not the same associations would be observed with MR scans obtained before the treatment. Second, this is an observational study and no causal relationship can be established between weight gain, impulsivity and brain structures. Third, the MRI technique for measuring changes in frontal and striatum volumes was different in cohort B (analyses on averaged volumes from selected ROIs), with an older population (median 56 years, IQR: 51–62) and with a long duration between weight measurements (median 63 months, IQR: 63–66), which differs from the other cohorts. Fourth, the assessments of impulsivity control scores did not rely on specific scales designed to measure this personality characteristic and differed between EPP and cohort B, with the former relying on a single PANSS item and the latter using the two dimensions of the NEO-FFI-R, which may account for differential findings between the two cohorts. Fifth, there were few plasma samples available for measuring MIF and PAI-1 levels in EPP. Although further studies using larger samples of patients with early psychosis are still needed, the current results may represent the first analysis of PAI-1 levels associated with weight changes induced by antipsychotics. To better understand the impact of treatment on weight gain, additional studies are required, including pre- and post-treatment comparisons.

The strength of the present study lies in the inclusion of patients with early psychosis and the availability of two additional cohorts with similar measures, which allowed us to test whether the findings derived from EEP can be generalized to unaffected individual or mostly untreated people from the general population. In addition, analyses in both population-based cohorts support the hypothesis that these differences in the fronto-striatal GM volumes, poor impulse control scores and in PAI-1 levels are only present in EPP and absent in the general population.

In summary, the present findings suggest that inter-individual variability in rapid weight gain induced by antipsychotics in EPP is associated with poor impulse control and with differences in brain regions related to impulsivity and is specific to the psychiatric population. Future research in this area, also confirming the observed association with the inflammatory marker PAI-1, may provide new insights into the underlying neurobiology of antipsychotic-induced weight gain.

### Supplementary information

Supplementary material

## Data Availability

Data from PsyMetab cannot be publicly deposited due to participant confidentiality purposes. Data from PsyMetab can be accessed after formal application and ethical review by the Ethics Committee of the Canton of Vaud. For further details: http://www.chuv.ch/cnp-psymetab. The dataset analyzed for the Treatment and Early Intervention in Psychosis Programme (TIPP) in the current study is available from the corresponding author upon reasonable request.
