# Supplementary material for: Associations between antipsychotics-induced weight gain and brain networks of impulsivity
Source: Transl Psychiatry. 2024 Mar 26;14:162. doi: 10.1038/s41398-024-02881-4 (PMC10965915; doi:10.1038/s41398-024-02881-4)
Supplement: Supplementary file 1 — Supplementary material [file 41398_2024_2881_MOESM1_ESM.docx]

**Supplementary Material**

Method

The scanner effect was removed from grey matter segmentations while maintaining the effects in age, gender and group differences^1^. The ComBat model, initially developed for gene expression analysis, was reformulated for use in the context of voxel-wise values. In this context, the data is assumed to originate from multiple imaging sites, each containing a varying number of scans. The model involves a location and scale adjustment framework, expressed as:

$$y_{ijv}=\alpha_{v}+X_{ij}\beta_{v}+\gamma_{iv}+\delta_{iv}\varepsilon_{ijv}$$

where $y_{ijv}$ denotes the grey matter value measure for voxel $v$ of scan $j$ at site $i$, $X$ is a design matrix for relevant covariates (eg. age and gender), and $\beta_{v}$ represents the voxel-specific vector of regression coefficients corresponding to $X$. The error terms $\varepsilon_{ijv}$ are assumed to follow a normal distribution with mean zero and variance $\sigma^{2}$. The terms $\gamma_{iv}$ and $\delta_{iv}$ signify the additive and multiplicative effects of site $i$ for voxel $v$.

ComBat estimates of the site-effect parameters $\gamma_{iv}^{*}$and $\delta_{iv}^{*}$ are computed using conditional posterior means, facilitating data analysis and interpretation in template space. The grey matter value for each voxel without scanner effect is finally given by the following expression:

$$y_{ijv}^{Combat}=\frac{y_{ijv}-\hat{\alpha}-X_{ij}\hat{\beta}-\gamma_{iv}^{*}}{\delta_{iv}^{*}}+\hat{\alpha}_{v}+X_{ij}\hat{\beta}_{v}$$

The effect of data harmonization using Combat (before and after values) can be observed elsewhere^2^. Individual T1-weighted images were also processed using FreeSurfer (v 6.0.0) to subdivide the gray matter in different regions of interest. This process encompassed generating precise cortical surfaces (white and pial) and identifying subcortical structures such as the caudate nucleus, putamen, nucleus accumbens, globus pallidum, amygdala, thalamus, hippocampus, and brainstem. The

surface reconstruction procedure was supervised and corrected by an operator who was blind to the subject's diagnosis when necessary. The cortical parcellation into

gyral-based regions of interest adhered to the anatomical criteria outlined in Desikan atlas ^3^, resulting in the generation of 34 cortical regions of interest for each hemisphere.

1. Fortin J-P, Parker D, Tunç B, et al. Harmonization of multi-site diffusion tensor imaging data. *Neuroimage*. 2017;161:149-170.

2. Alemán-Gómez Y, Baumgartner T, Klauser P, et al. Multimodal magnetic resonance imaging depicts widespread and subregion specific anomalies in the thalamus of early-psychosis and chronic schizophrenia patients. *Schizophrenia Bulletin*. 2023;49(1):196-207.

3. Desikan RS, Ségonne F, Fischl B, et al. An automated labeling system for subdividing the human cerebral cortex on MRI scans into gyral based regions of interest. *Neuroimage*. 2006;31(3):968-980.

**Supplementary Table 1. Comparison between early psychosis patients and cohort A**

|  | **EPP**  **(N=42)** | **Cohort A**  **(N=102)** | ***P^1^*** |
| --- | --- | --- | --- |
| **Women, N (%)** | 15 (36%) | 39 (38%) | 0.92 |
| **Age median (IQR), years** | 23 (21-27) | 26 (22-33) | **0.002** |
| **BMI during scan (IQR), kg/m²** | 24 (22-27) | 23 (21-24) | **0.008** |
| **Years of education median (IQR), years** | 13 (10-15) | 16 (13-17) | **<0.001** |
| **Smoking, N (%)*^2^*** | 25 (59%) | 8 (8%) | **<0.001** |
| **Cannabis users, N (%)** | 12 (29%) | 5 (5%) | **<0.001** |

Data are medians (IQR) or numbers (percentage).

*^1^P*-values for statistical comparisons between EPP/cohort A, Wilcoxon-Mann-Whitney rank-sum tests for continuous variables and chi-square test for categorical variable; *P*<0.05 in bold.

*^2^*Data were not available for 2 out of 102 participants in the cohort A.

BMI: body mass index

EPP: early psychosis patients

IQR: interquartile range

**Supplementary Table 2. Demographic and clinical characteristics of the participants in the cohort A**

|  | **Normal BMI <25kg/m² (N=83)** | **High BMI ≥25kg/m² (N=19)** | ***P^1^*** |
| --- | --- | --- | --- |
| **Women, N (%)** | 35 (42%) | 4 (21%) | 0.15 |
| **Age median (IQR), years** | 26 (22-32) | 32 (26-36) | **0.03** |
| **BMI median (IQR), kg/m²** | 22 (21-23) | 26 (25-27) | **<0.001** |
| **Years of education median (IQR), years** | 15 (13-17) | 17 (15-20) | **0.019** |
| **Smoking, N (%)*^2^*** | 7 (8%) | 5 (5%) | 1 |
| **Cannabis users, N (%)** | 5 (6%) | 0 (0%) | 0.77 |

Data are medians (IQR) or numbers (percentage %).

*^1^P*-values for statistical comparisons between groups, Wilcoxon test for continuous variables and chi-square test for categorical variable; *P*<0.05 in bold.

*^2^*Data were not available for 2 out of 102 participants with normal BMI.

BMI: body mass index

IQR: interquartile range

**Supplementary Table 3. Demographic and clinical characteristics of the participants in the cohort B**

|  | **HW_cohortB_**  **(N=146)** | **LW_cohortB_**  **(N=729)** | **P*^1^*** |
| --- | --- | --- | --- |
| **Women, N (%)** | 88 (60%) | 355 (49%) | **0.014** |
| **Age median (IQR), years** | 48 (44-53) | 51 (46-57) | **<0.001** |
| **BMI during F2, median (IQR), kg/m²** | 29 (26-32) | 25 (23-28) | **<0.001** |
| **Years of education, median (IQR), years** | 14 (12-16) | 14 (12-17) | 0.73 |
| **Smoking, N (%)*^2^*** | 80 (55%) | 387 (53%) | 0.27 |
| **Duration between F1–F2, median (IQR), days** | 1900 (1900-2000) | 1900 (1900-2000) | 0.85 |

Data are medians (IQR) or numbers (percentage) for the participants with high weight gain and those with low weight gain between follow up 1 and follow up 2.

*^1^P*-values for statistical comparisons between the high weight gain group (HW**_cohortB_**) if they had a weight gain of ≥7% or more during the period between the first and second follow-up, and the low weight gain group (LW**_cohortB_**) if they gained <7%. Wilcoxon test for continuous variables and chi-square test for categorical variable; *P*<0.05 in bold.

*^2^*Data were not available for 29 out of 729 participants with low weight gain and 7 out of 146 participants with high weight gain.

BMI: body mass index

CI: confidence interval

F1: follow-up 1

F2: follow-up 2

IQR: interquartile range

|  | **Frontal lobe*^1^*** | | | **Striatum*^1^*** | | |
| --- | --- | --- | --- | --- | --- | --- |
| ***Predictors*** | ***β*** | ***95% CI*** | ***P*** | ***β*** | ***95% CI*** | ***P*** |
| **(Intercept)** | 0.18 | 0.13 – 0.24 | **<0.001** | 0.17 | 0.13 – 0.23 | **<0.001** |
| **Age (years)** | -0.44 | -0.50 – -0.37 | **<0.001** | -0.06 | -0.07 – -0.05 | **<0.001** |
| **Women** | 0.75 | -0.39 – 1.89 | 0.20 | 0.30 | 0.10 – 0.50 | **0.003** |
| **TIV (cm^3^)** | 0.07 | 0.07 – 0.08 | **<0.001** | 0.01 | 0.01 – 0.01 | **<0.001** |
| **Low Weight Gain group** | -0.66 | -1.87 – 0.55 | 0.28 | 0.12 | -0.09 – 0.33 | 0.27 |

**Supplementary Table 4.** **Generalized regression model between weight gain groups and impulsivity associated with brain regions in cohort B (N=875)**

Estimates with 95% confidence intervals and *P*-values are reported from linear regression models. *P*<0.05 in bold.

*^1^*Frontal lobe and striatum volumes are expressed in cm^3^.

*β*: beta coefficient

CI: confidence interval

TIV: total intracranial volume

**Supplementary Table 5**. **Linear models for MIF and PAI-1 levels in cohort A (N=102)**

|  | **MIF (ng/mL)** | | | **PAI-1 (U/mL)** | | |
| --- | --- | --- | --- | --- | --- | --- |
| ***Predictors*** | ***β*** | ***95% CI*** | ***P*** | ***β*** | ***95 % CI*** | ***P*** |
| **(Intercept)** | 40 | -48 – 127 | 0.36 | -11 | -47 – 25 | 0.52 |
| **Age (years)** | 1.1 | -0.79 – 3.0 | 0.25 | 0.33 | -0.43 – 1.1 | 0.39 |
| **Women** | -12 | -30 – 5.5 | 0.17 | -1.7 | -9.0 – 5.6 | 0.62 |
| **BMI (kg/m^2^)** | -0.34 | -4.0 – 3.3 | 0.85 | 0.56 | -0.95 – 2.1 | 0.46 |

Estimates with 95% confidence intervals and *P*-values are reported from linear regression models. *P*<0.05 in bold.

*β*: beta coefficient

BMI: body mass index

MIF: macrophage migration inhibitory factor

PAI-1: plasminogen activator inhibitor-1

CI: confidence interval

**Supplementary Figure 1. Resting State Functional Connectivity between the right primary motor cortex and the pallidum in function of weight gain (%) in early psychosis patients**


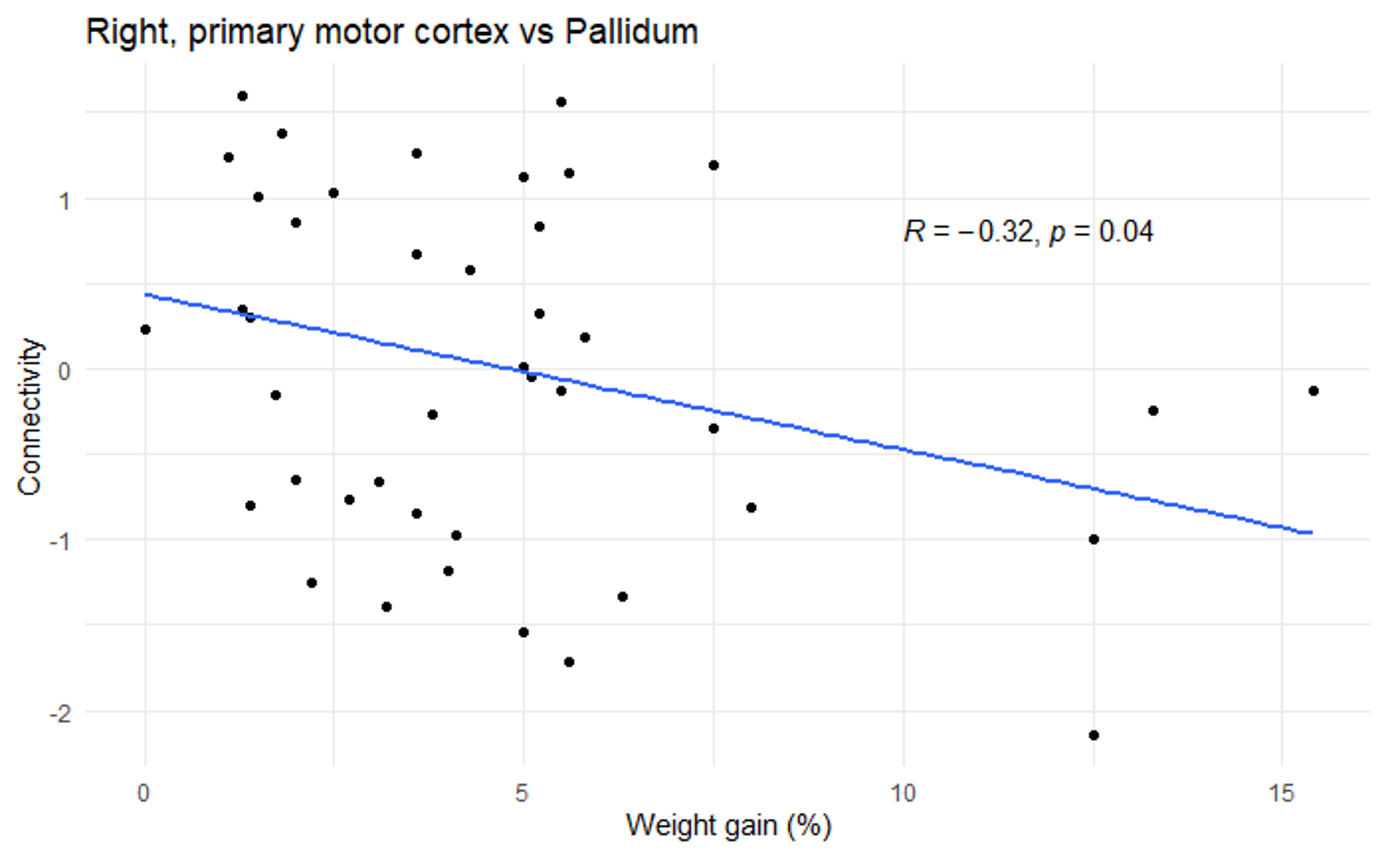


Scatterplot illustrating the association between the resting state functional connectivity (RSFC) and weight gain (%) measured in early psychosis patients, after one month of treatment at risk of inducing weight gain (N=42). Pearson’s correlation coefficient indicated a negative correlation between RSFC between the right primary motor cortex and the right pallidum (*R*=-0.32, *P*=0.04).
